# Supplementary material for: Cold-Azurin, a New Antibiofilm Protein Produced by the Antarctic Marine Bacterium Pseudomonas sp. TAE6080
Source: Mar Drugs. 2024 Jan 25;22(2):61. doi: 10.3390/md22020061 (PMC10890351; doi:10.3390/md22020061)
Supplement: Supplementary file 1 [file marinedrugs-22-00061-s001.zip › marinedrugs-2825560-supplementary.pdf]

Supplementary:

```
AZ6080             1 mfakivavslltlasgqllaaeckvtvdstdqmsfdtkaieidkscktft     50
                       |..|:.|||||:|.|..||||||.|.:...|||.|:|.||.:|||||.||
BSEQ0021295        1 MLRKLAAVSLLSLLSAPLLAAECSVDIQGNDQMQFNTNAITVDKSCKQFT     50

AZ6080            51 vdlkhsgnlpknvmghnwvltteadmqpvategmaagidknylkegdtri    100
                       |:|.|.||||||||||||||:|.||||.|.|:|||:|:||:|||..|:|:
BSEQ0021295       51 VNLSHPGNLPKNVMGHNWVLSTAADMQGVVTDGMASGLDKDYLKPDDSRV    100

AZ6080           101 iahtkiigagetdsvtfdvsklkadgkymffcsfpghismmkgtvtlk      148
                       |||||:||:||.|||||||||||...:|||||:||||.::||||:|||
BSEQ0021295      101 IAHTKLIGSGEKDSVTFDVSKLKEGEQYMFFCTFPGHSALMKGTLTLK      148
```

Figure S1. Comparison of *Pseudomonas sp* TAE6080 and *Pseudomonas aeruginosa* PAO1 Azurin. Sequence alignment of the amino acid sequences of *Pseudomonas sp.* TAE6080 Cold-Azurin (Az6080) and the Azurin produced by *Pseudomonas aeruginosa* PAO1 (BSEQ0021295) revealed an identity of (68.2%) and a similarity of (81.8%)

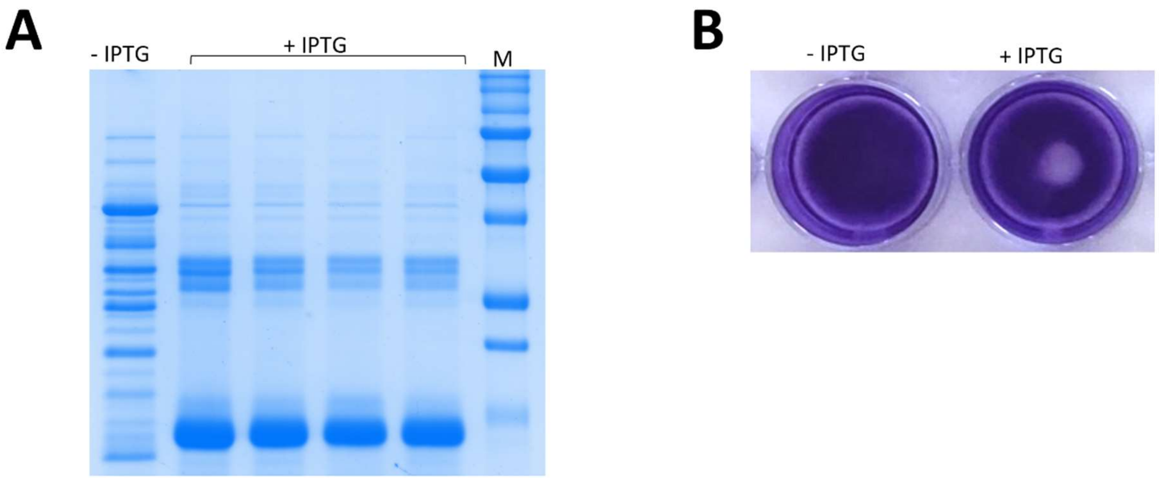

Figure S2. Protein profile and anti-adhesive activity of periplasmic fraction from recombinant *E. Coli* BL21DE3. (A) SDS-PAGE 15% stained with Comassie blue, protein profile of periplasmic fraction from recombinant *E. Coli* BL21DE3 induced (+ IPTG) or non-induced -IPTG) cells. .M: molecular weight marker. (**B**) Biofilm formation by *S. epidermidis* O-47 in polystyrene 24-wells microtiter plate wells coated with periplasmic fraction from recombinant *E. Coli* BL21DE3 induced (+ IPTG) or non-induced (-IPTG) cells.

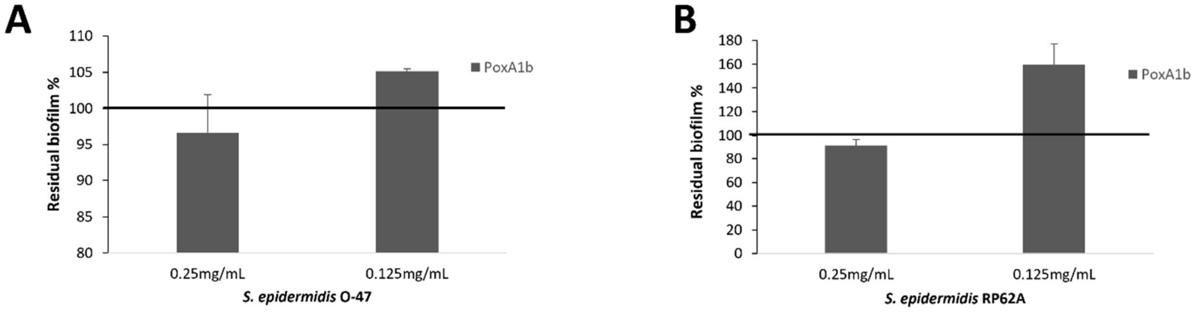

Figure S3. Antibiofilm activity of poxA1b laccase. Laccase (PoxA1b) antibiofilm activity against *S. epidermidis* O-47 (**A**) or *S. epidermidis* RP62A (**B**). Concentrations shown in the assay (0.25 mg/mL or 0.125 mg/mL) correspond to 3.75 U/mL or 1.87 U/mL of poxA1b used for the experiment.Each data point represents the mean

± SD of four independent samples. The results are expressed as the percentage of biofilm formed in the presence of laccase compared to untreated bacteria (100%). Biofilm formation was considered unaffected in the range of 90–100%.
